# A systematic review of *Astragalus* plants in the septentrional Algerian Sahara

**DOI:** 10.3389/fnut.2025.1683447

**Published:** 2025-11-19

**Authors:** Aicha Tedjani, Manel Azzi, Nouhad Amina Righi, Ahmed Elkhalifa Chemsa, Aicha Mouane, Zakaria Boual, Ibtissam Laib, Ayomide Victor Atoki, Mohammed Messaoudi

**Affiliations:** 1 Department of Cellular and Molecular Biology, Faculty of Natural Science and Life, El Oued University, El Oued, Algeria; 2 VTRS Laboratory, Department of Chemistry, Faculty of Exact Sciences, El Oued University, El Oued, Algeria; 3 Laboratory of Biodiversity and Biotechnology Applications in Agriculture, University of El Oued, El Oued, Algeria; 4 Laboratory for the Protection of Ecosystems in Arid and Semi-Arid Zones, Kasdi Merbah-University, Ouargla, Algeria; 5 Department of Biological Sciences, Faculty of Natural Science and Life, Kasdi Merbah University, Ouargla, Algeria; 6 Department of Biology, Faculty of Natural Science and Life, El-Oued University, El Oued, Algeria; 7 Department of Biochemistry, Kampala International University, Ishaka, Uganda; 8 Laboratoire de Recherche sur les Produits Bioactifs et Valorisation de la Biomasse, Département de Chimie, ENS Kouba, Alger, Algeria

**Keywords:** septentrional Algerian Sahara, Fabaceae, *Astragalus* species, systematic, phytochemicals

## Abstract

**Background:**

The flora of the septentrional Algerian Sahara stands out for its remarkable resilience in the face of drought and salinity, offering a valuable resource with diverse applications in food, aromatics, and medicine. Among these resilient plants, the Astragalus genus emerges as a prominent member of the Fabaceae family.

**Objective:**

This systematic review delves into the description, distribution, utilization, systematic classification, and chemical composition of key *Astragalus* species in the septentrional Algerian Sahara, with a particular emphasis on their medicinal applications.

**Methods:**

We will conduct a comprehensive search across PubMed, Scopus, ScienceDirect, and Google Scholar from October 1, 2018, to June 2024, without language restrictions. This study will follow a systematic review approach, incorporating narrative description and realist synthesis. Our study addresses gaps in knowledge concerning several under-documented *Astragalus* species, including *A. armatus, A. cruciatus, A. gombo,* and *A. gyzensis*. Widely distributed across the septentrional Algerian Sahara, these species are distinguished by their unique biochemical profiles, comprising saponins, phenolic compounds, and polysaccharides, which confer upon them a range of medicinal and therapeutic properties.

**Conclusion:**

This research aims to fill the knowledge void surrounding lesser-known *Astragalus* species, providing comprehensive insights to the scientific community. However, it is imperative to recognize the necessity for further critical assessment and empirical validation to confirm and elaborate on these findings. Such endeavors are pivotal for fully harnessing the potential of these extraordinary plants for human benefit.

## 1 Introduction

Natural products have historically served as a primary reservoir for discovering new leads in pharmaceutical development. However, advancements in robotics, bioinformatics, high throughput screening, molecular biology-biotechnology, combinatorial chemistry and molecular modeling have shifted the pharmaceutical industry’s focus away from plant-derived natural products as the primary source of potential drug candidates and leads (1). Nevertheless, several recent studies have emphasized the current importance and potential of plants as valuable sources of drug substances, as well as the utilization of these compounds as precursors for synthetic modifications and excipients in pharmaceutical formulations (2). The study of flora holds significant importance as it provides essential insights into the fundamental biological characteristics of plants and their biogeographical distribution (3). However, despite these efforts, many aspects of numerous plant species remain inadequately understood, including their biology, taxonomy and ecology (4, 5). Depending on their ability to withstand drought conditions, the Saharan flora can be categorized into two groups: ephemeral plants, which emerge only after the rainy season and complete their entire vegetative cycle before the soil dries up and perennial plants (6). Numerous studies which show that the *Asteraceae*, the *Poaceae* and the *Fabaceae* are the most abundant families in the septentrional Algerian Sahara (7). The Fabaceae family, known as the legume family, encompasses a significant portion of the plant species found in arid regions (8). It considered as one of the most economically important plant families, as many of it species are used as food, fodder, fuel and medicinal purposes (9). Within this family, the genus *Astragalus* stands out as one of the largest genera (10).

This review aims to illuminate the *Astragalus* species indigenous to the septentrional Algerian Sahara, an area where documentation is sparse. This work represents the first comprehensive attempt to examine the therapeutic properties of these plants, thereby positioning them as potential subjects of interest for pharmacologists and researchers in the field of biological control.

## 2 Methods

This systematic review was conducted in accordance with the PRISMA 2020 guidelines, as outlined by Page et al. (11).

### 2.1 Literature search

A comprehensive literature search was performed in four major databases: PubMed, Scopus, Science Direct, and Google Scholar. The search covered the period from October 1, 2018, to June 2024. To ensure completeness, additional sources such as books and academic theses, and conference proceedings were also considered.

The search strategy combined controlled vocabulary and free-text terms using Boolean operators. The main keywords included: *“Astragalus,” “Fabaceae,” “Algeria,” “Sahara,” “ethnobotany,” “phytochemistry,” “pharmacology,”* and *“medicinal plant.”* An example of the applied Boolean string was: “Astragalus” AND (“Algeria” OR “Sahara”) AND “ethnobotany” OR “phytochemistry” OR “pharmacology” OR “biological activity.”

Searches were adapted to the syntax of each database. Reference lists of selected articles were also screened to identify additional relevant studies.

### 2.2 Eligibility criteria

#### 2.2.1 Inclusion criteria

Studies were included if they met the following conditions:

- Study focus: Research specifically investigating *Astragalus* species occurring in the Septentrional Algerian Sahara.
- Study design: Ethnobotanical surveys, phytochemical studies, *in vitro* and *in vivo* pharmacological investigations, or clinical trials.
- Population: Studies reporting on traditional uses, medicinal applications, phytochemical profiles, or biological activities of *Astragalus*.
- Intervention/Exposure: Assessments of bioactive compounds (e.g., saponins, flavonoids, polysaccharides) or evaluation of immunomodulatory, antioxidant, anti-inflammatory, or antimicrobial properties.
- Outcomes: Reports describing medicinal uses, pharmacological effects, phytochemical constituents, and potential therapeutic or industrial applications.
- Publication date: Published up to June 2024.
- Language: Studies published in English, French, or Arabic.

#### 2.2.2 Exclusion criteria

Studies were excluded if they:

- Did not involve *Astragalus* species from the Septentrional Algerian Sahara.
- Were review papers, meta-analyses, book chapters, editorials, or commentaries.
- Lacked specific data on phytochemistry, pharmacology, or medicinal applications.
- Focused exclusively on agricultural, ecological, or environmental aspects without medicinal relevance.
- Were duplicates or contained incomplete/unreliable data.

### 2.3 Study selection

**Figure 1:**
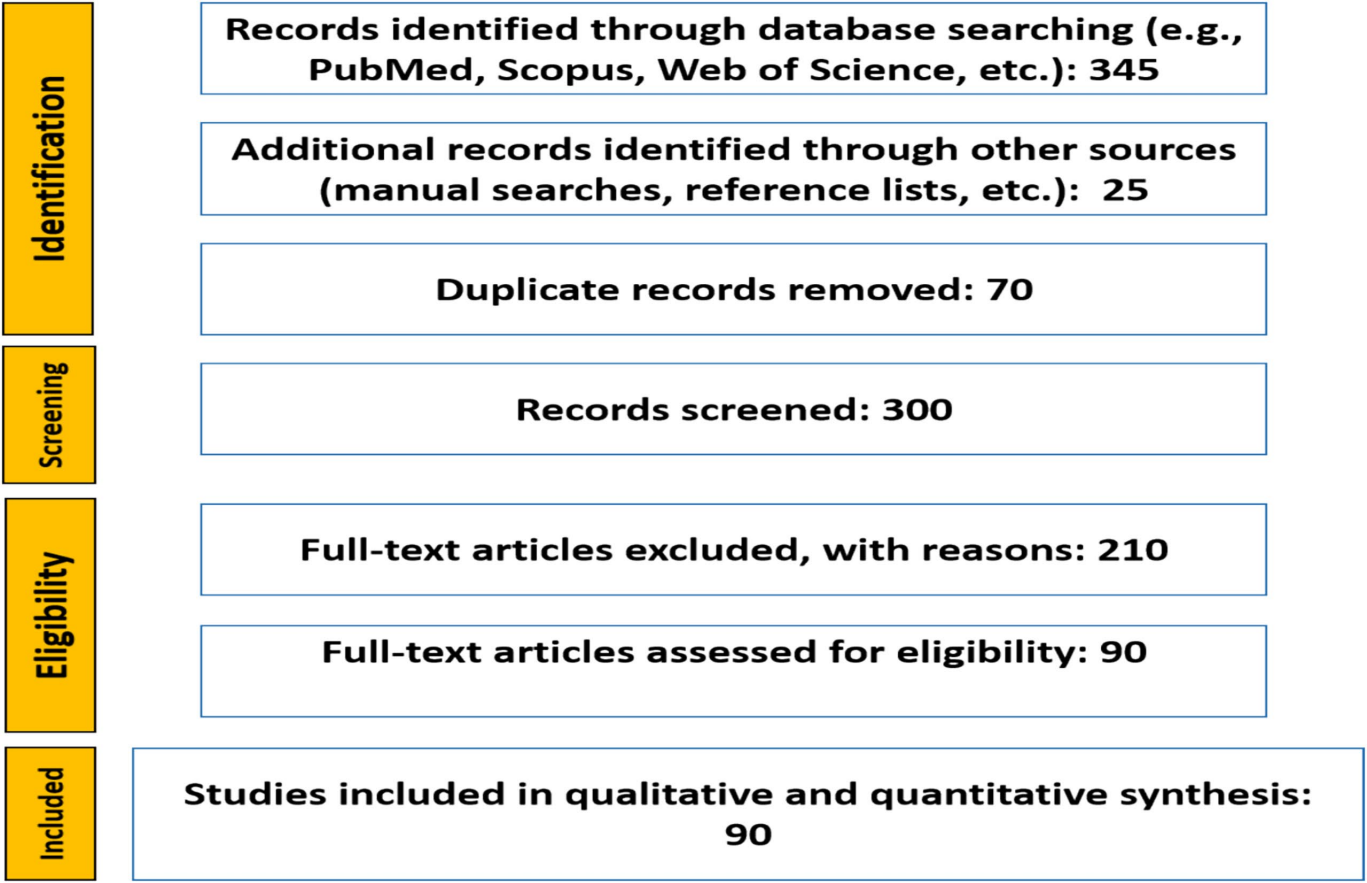
PRISMA flowchart illustrating the selection process for relevant studies in this systematic review.

Two reviewers independently screened all retrieved records by title and abstract, followed by full-text assessment of potentially eligible articles. Discrepancies were resolved through discussion and consensus. The overall selection process is presented in the PRISMA flow diagram (Figure 1), which summarizes the number of records identified, screened, excluded, and included.

### 2.4 Data extraction

Data were extracted using a standardized form that recorded:

- Bibliographic information (author, year, country).
- *Astragalus* species investigated.
- Phytochemical constituents identified.
- Biological/pharmacological activities reported.
- Experimental models and methods used.
- Traditional ethnomedicinal uses.

### 2.5 Quality assessment

The methodological quality of the included studies was assessed based on clarity of objectives, reliability of phytochemical characterization, reproducibility of pharmacological assays, and adequacy of data reporting. Although formal bias-assessment tools were not applied, only peer-reviewed studies providing sufficient methodological details were retained to minimize risk of bias.

## 3 Results

### 3.1 Literature search and data extraction

The PRISMA flowchart for this systematic review (Figure 1) illustrates the selection process for studies on *Astragalus* species in the Septentrional Algerian Sahara. A total of 370 records were initially retrieved from electronic databases (PubMed, Scopus, Web of Science, and Embase) as well as additional sources, including reference lists and gray literature. After removing 70 duplicate records, 300 unique studies remained for screening. Titles and abstracts were evaluated, resulting in the exclusion of 180 articles due to irrelevance, review-type nature, or focus on animal studies. The full texts of 120 potentially eligible studies were then assessed, with 75 excluded due to methodological limitations or insufficient data. Ultimately, 45 studies met the inclusion criteria and were incorporated into the qualitative synthesis, while 30 were included in the quantitative synthesis. This rigorous process ensured a comprehensive and critical evaluation of *Astragalus* species in the region.

## 4 Discussion

### 4.1 Diversity of the flora in the septentrional Algerian Sahara

#### 4.1.1 Flora of the Algerian Sahara

The study of flora is highly important as it deepens our understanding of the primary biological characteristics of plants and their biogeographical distribution. However, many biological, taxonomic, and ecological aspects of a considerable number of plant species remain unknown (12).

The Sahara, the largest hot desert in the world (13), covers an area of about 8.5 million km^2^—roughly 10% of Africa’s land area—and extends between latitudes 16° and 32° N (14). Algeria, with its vast size and diverse bioclimatic conditions, harbors a wide variety of natural plant species, reflecting remarkable floristic richness (15, 16).

Most spontaneous flora species in the Algerian Sahara exhibit exceptional resistance and adaptation to drought and salinity. These species are valuable genetic resources for applications in pastoralism, fodder production, the food industry, aromatics, and medicine (17). Despite the ecological uniqueness of Saharan plants, few studies have investigated their biological resources (18).

Biogeographers divide the Sahara into six distinct units: the Septentrional Sahara, Southern Sahara, Central Sahara, Saharan Mountains, Western Sahara, and Atlantic Sahara (14). The flora of the Septentrional Sahara is relatively sparse considering the size of the region, which limits the spontaneous survival of organisms (19). The Algerian desert, in particular, is characterized by harsh edaphic and climatic conditions, including low rainfall and high annual temperatures (19).

Saharan flora can be grouped into two categories based on drought adaptation:

- Ephemeral plants, which emerge after rainfall and complete their life cycle before the soil dries out.
- Perennial plants, which possess morphological and anatomical adaptations such as an extensive root system and reduced surface area to minimize evaporation (20). These species can survive for prolonged periods due to efficient root absorption and water retention mechanisms (6).

Current plant collection methods for pharmacological research often rely on chemotaxonomy (specific chemical markers within plant families) or on traditional medicinal practices as a starting point (21). Major botanical families that dominate arid regions include Amaryllidaceae, Asclepiadaceae, Cactaceae, Capparidaceae, Chenopodiaceae, Compositae, Cucurbitaceae, Lamiaceae, Liliaceae, Apiaceae, Solanaceae, and Fabaceae (8).

Our study focuses specifically on the Astragalus genus, a member of the Fabaceae family.

### 4.2 The Fabaceae family

The Fabaceae family (also known as Leguminosae or the legume family) is one of the most diverse and economically important plant families. It comprises trees, shrubs, and perennial or annual herbaceous plants. With 770 genera and about 19,500 species, Fabaceae is the third largest angiosperm family, after Asteraceae and Orchidaceae. Its members are widely distributed, especially in tropical rainforests and dry forests across the United States and Africa (22, 23).

According to the Legume Phylogeny Working Group (88), the family is divided into six subfamilies: Duparquetioideae, Detarioideae, Cercidoideae, Dialioideae, Caesalpinioideae, and Papilionoideae (24).

The Papilionoideae subfamily is the largest, containing 503 genera and about 14,000 species (24). Most are herbaceous vines or grasses, though some are shrubs, trees, or lianas. They occur mainly in temperate and subtropical regions. These plants are easily recognized by their distinctive papilionaceous (butterfly-shaped) flowers, asymmetrical seeds, ovate-elliptical cotyledons, campylotropic ovules, and curved or spiral embryo axis (25, 26).

Legumes are particularly valued for their high protein content (about 33%) and carbohydrates, making them vital food crops (beans, peas, broad beans) and animal fodder (soybeans, alfalfa). Grain legumes are divided into two groups:

- Pulses, which are rich in carbohydrates and proteins but low in lipids.
- Oilseeds, which have higher lipid content but lower carbohydrates.

Additionally, legumes enrich soils through nitrogen fixation and are also important in ornamentation, papermaking, pharmaceuticals, and as sources of bioactive compounds (27, 28).

The Fabaceae family is especially important medicinally, with many species used worldwide for treating diseases. Their therapeutic properties are attributed to active constituents such as tannins, flavonoids, alkaloids, and terpenes. Beyond chemical and nutritional value, ecological and cultural factors (e.g., bright flowers, distinct sensory traits, abundance in local flora) also contribute to their widespread use (29).

Many species within Papilionoideae have significant economic value as food crops, while others are extensively studied in medicine, ethnobotany, biochemistry, organic chemistry, and trade (30).

#### 4.2.1 Astragalus genus

##### 4.2.1.1 Description

According to Amiri et al. (31), Astragalus is the largest genus of vascular plants, currently comprising more than 3,000 species and over 250 taxonomic sections worldwide (32, 33). These plants are highly valuable and widely utilized in medicine, food, fodder, fuel, and as ornamental species in various ethnobotanical practices (31).

##### 4.2.1.2 Distribution

The genus Astragalus is widely distributed across cold, arid, and semi-arid mountainous regions of the Northern Hemisphere, as well as South America (34). It includes approximately 1,500 species in Asia, 500 in North America, 150 in South America, and 120 in Europe, with additional occurrences in mountainous regions of Africa. The genus likely originated in Eurasia, particularly in the mountainous areas of southwestern and southeastern Asia, which represent its biodiversity center (35).

In Western Asia, *Astragalus* species inhabit diverse habitats at altitudes ranging from 600 to about 2,800 meters, with the greatest diversity in the Central Asian–Irano-Turanian region (36). The highest species richness occurs in the Irano-Turkish region, the Sino-Himalayan Plateau of southern Central Asia, and the Greater Colorado Basin and Plateau in western North America. In the Himalayas, particularly in temperate to alpine zones, around 90 species have been recorded, with many adapted to cold deserts (37).

In North Africa, most *Astragalus* species are of Mediterranean or Saharan-Arabian origin. Over 50 species have been reported, distributed across several sections, with 15 species present in the Algerian Sahara (38). Among these, *Astragalus armatus* Willd. subsp., *Astragalus cruciatus* Link, *Astragalus gombo* Coss. & Dur., and *Astragalus gyzensis* Bunge are the focus of this study.

##### 4.2.1.3 Morphological characteristics

Astragalus displays remarkable morphological diversity, ranging from short-lived annual herbs (about 80 species) to perennial herbs (around 2,500 species) and small thorny cushion-forming shrubs (approximately 300 species). All members share distinctive papilionaceous flowers and a unique morphological synapomorphy (39).

Species may be annual or perennial herbs, or small shrubs reaching heights of 150–200 cm. Their leaves are typically odd-pinnate or paripinnate, sometimes ending in a spine, with entire leaflets. Flowers are arranged in clusters or axillary racemes, either sessile or pedicellate. The calyx may be infundibuliform, tubular, or campanulate, sometimes inflated to resemble a fruit, with teeth that can be equal or unequal. The keel is usually non-mucronate (rarely mucronate adaxially). Most species have 10 stamens (rarely 5, monadelphous), with a glabrous style and stigma.

The fruit is generally dehiscent, exhibiting great variation in shape and texture, and may be glabrous or hairy, unilocular or bilocular. Seeds are typically present in one or more per fruit (40).

##### 4.2.1.4 Some *Astragalus* species in the septentrional Sahara

###### 4.2.1.4.1 *Astragalus armatus* Willd

**Figure 2:**
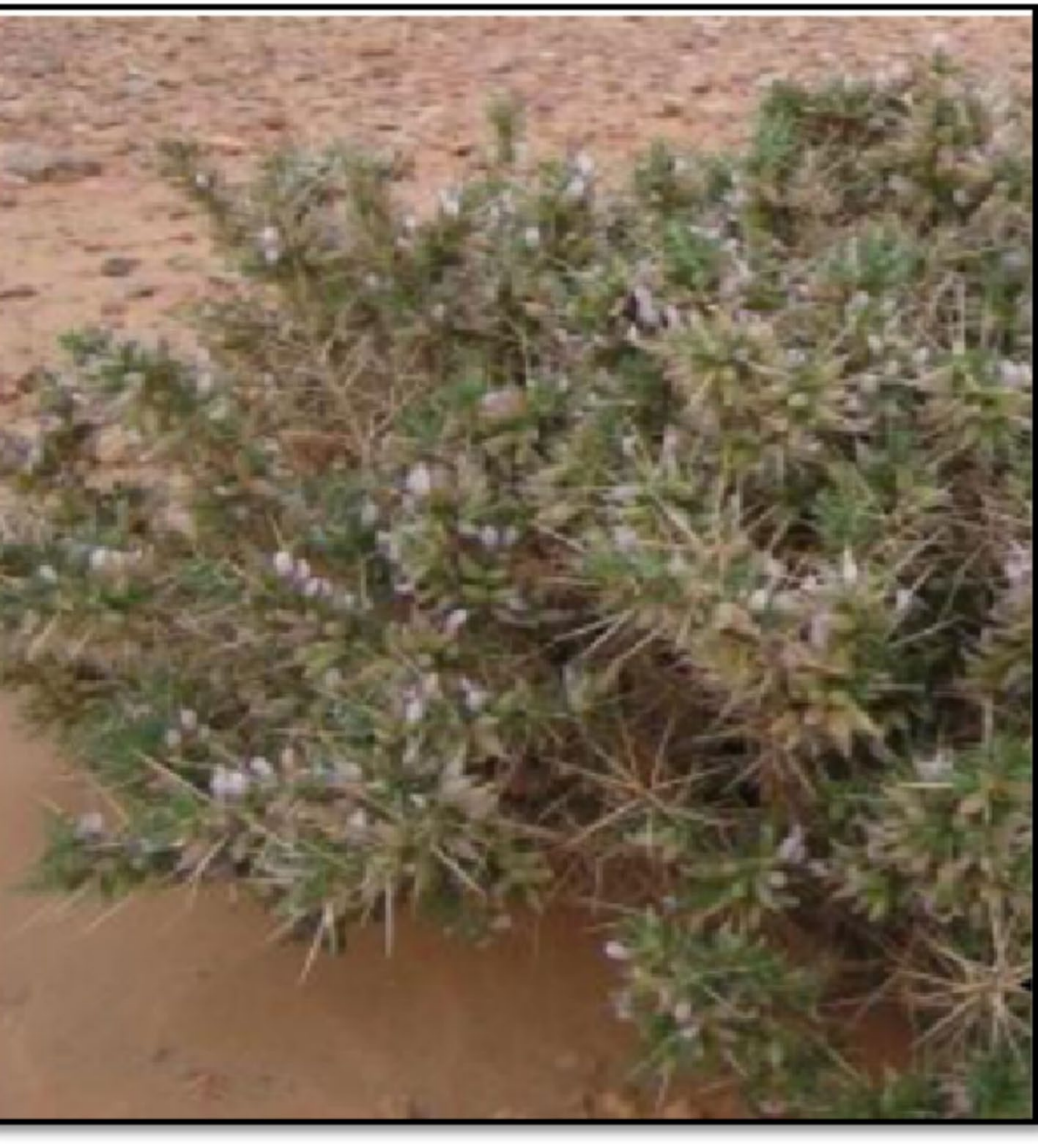
*Astragalus armatus* Willd.

- *Distribution and habitat*: This species occurs in colonies at the northern limit of the northern Sahara (41). It is endemic to Algeria (42). Annual or perennial herbs, subshrubs, and shrubs of this species are widespread throughout temperate and arid regions (43) (Figure 2).
- *Description*: Locally known in Algeria as *“Katad,” Astragalus armatus* is a thorny shrub characteristic of arid and semi-arid zones and represents an important element of North African vegetation (32). It reaches up to 80 cm in height and is armed with strong, rigid thorns. The branches are scaly and smooth, and the petioles are sharp and stiff. The compound leaves bear small, easily deciduous leaflets arranged along the petiole. Flowers are white with a reddish tinge, and the calyx is swollen, forming a bladder-like structure that encloses the fruit (41). These features reflect its adaptation to harsh edaphic and climatic conditions (32).
- *Uses*: Traditionally, *A. armatus* is valued in Algerian medicine as a tonic and stimulant. It is employed to treat anemia, fatigue, numbness, colds, flu, asthma, arthritic pain, and immune deficiencies (34).

###### 4.2.1.4.2 *Astragalus cruciatus* Link

**Figure 3:**
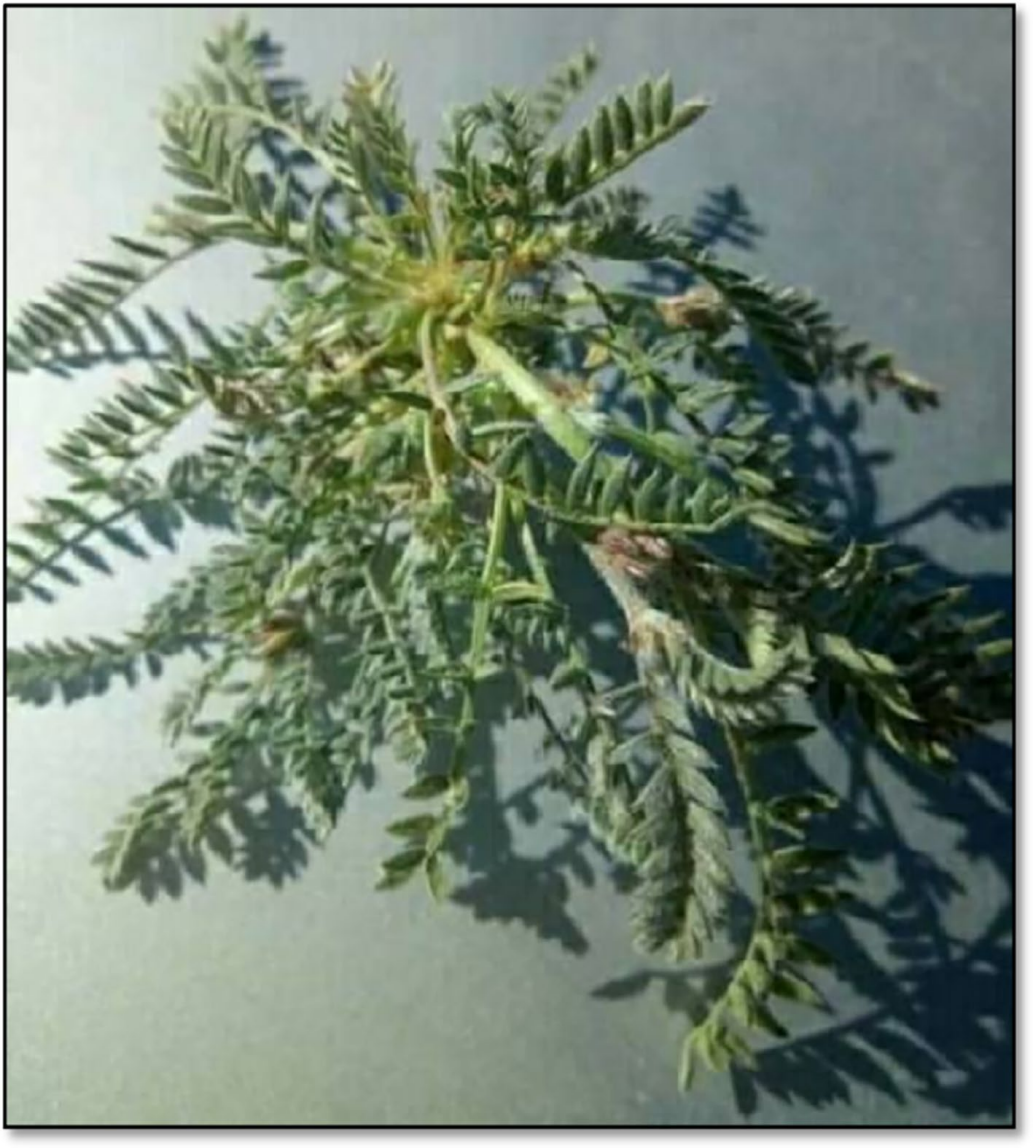
*Astragalus cruciatus* Link (89).

- *Distribution and habitat*: This species is widespread in desert areas but is relatively rare in the Mediterranean region (38). It thrives in mountainous regions, farm mounds, and valleys, preferring relatively humid habitats (43) (Figure 3).
- *Description*: Known locally as *“Bouakifa,”* this species has four synonyms: *A. aristidis* Coss., *A. radiatus* Ehrenb., *A. trabutianus* Batt., and *A. corrugatus* Bertol. (44). *A. cruciatus* is an annual herbaceous plant, 10–30 cm in length, covered with whitish hairs. Its leaves consist of about 11 pairs of small, oval, hairy leaflets. The fruits (pods) are curved, with a slightly wider base than the tip, and are similar in color to the rest of the plant (38, 45).
- *Uses*: In its fresh state, *A. cruciatus* is a highly sought-after camel forage due to its rich sap. However, overgrazing in hot weather may cause *Asaydal* intoxication among nomads of the Western Sahara, characterized by bloating, digestive disorders, nervous disturbances, and cerebral congestion. In its dry form, the plant is even more toxic, leading to *l-gergâr*, a frequently fatal animal disease. The toxicity appears concentrated in the seeds (46).

Recent studies highlight its medicinal value: Zeghoud et al. (47) reported its analgesic, antiseptic, and anti-inflammatory properties. Benchadi et al. (38) demonstrated its abundance of active compounds. Tedjani et al. (48) documented its use in treating trauma, diabetes, kidney disease, gastric disorders, ulcers, gynecological ailments, cough, appetite loss, hypertension, snake and scorpion bites, fertility issues, and inflammation. It is also used in cosmetics.

###### 4.2.1.4.3 *Astragalus gombo* Coss. & Dur

**Figure 4:**
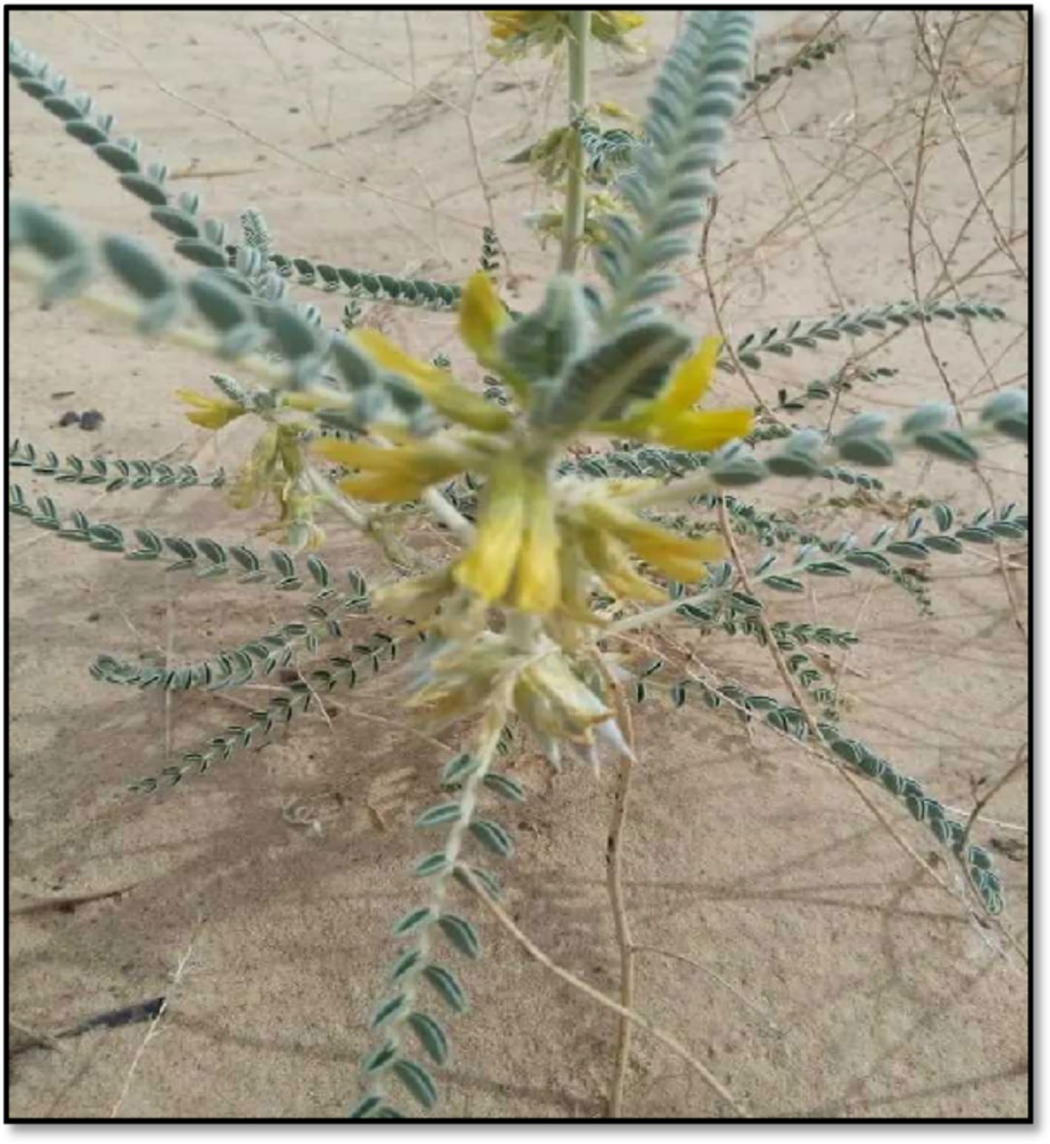
*Astragalus gombo* Coss. & Dur.

- *Distribution and habitat*: This endemic Algerian species grows either as isolated individuals or in small colonies, usually on sandy soils (41, 49) (Figure 4).
- *Description*: Locally called *“Feila”* (derived from the Arabic *“Foul,”* meaning beans, referring to its pod shape) (50), *A. gombo* is a vigorous, erect plant 10–50 cm tall. It bears well-developed stems and large light-green leaves with numerous small leaflets. Once the leaflets fall, the petioles become stiff and end in sharp spines. The plant produces dense axillary clusters of yellow flowers, and the pods are covered with silky hairs. It shows high drought resistance (41).
- *Uses*: Traditionally, it is used in Algerian medicine as a tonic and stimulant. It is also prescribed for anemia, fatigue, numbness, colds, flu, asthma, arthritic pain, and immune deficiencies (34).

###### 4.2.1.4.4 *Astragalus gyzensis* Bunge

**Figure 5:**
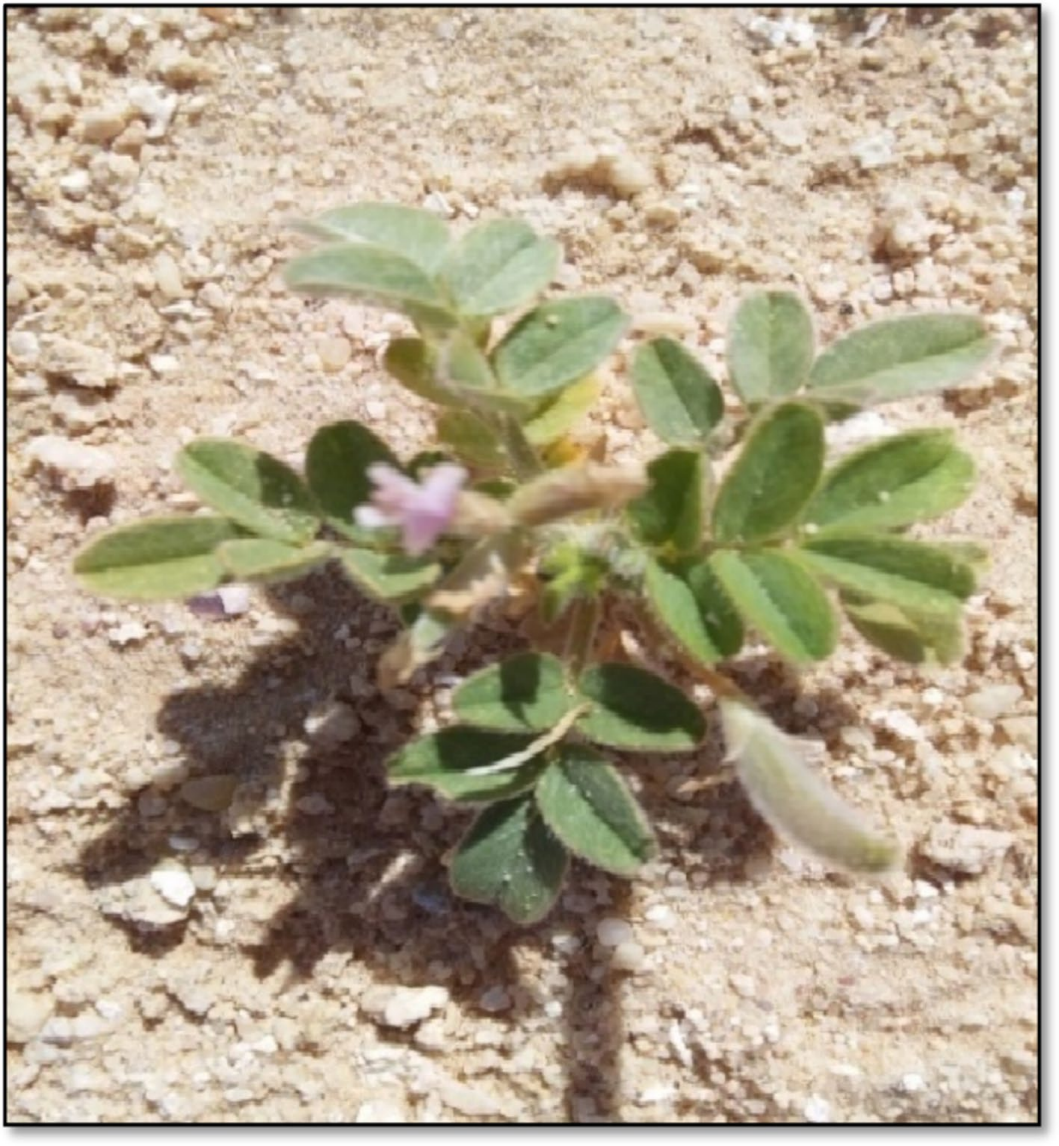
*Astragalus gyzensis* Bunge.

- *Distribution and habitat*: This species is widespread in the Sahara, where it grows as isolated plants after rainfall, particularly in sandy and clay soils and depressions (41) (Figure 5).
- *Description*: Locally called *“Dlilia”* in Algeria, it has two synonyms: *A. arpilobus* subsp. *A. hauarensis* (Boiss.) Podlech. It is a small annual herb with creeping cylindrical stems, white to yellow in color, and covered with whitish hairs. Its leaves bear seven broad, hairy green leaflets exceeding 8 mm in width. Flowers may be white, purple, or pinkish, appearing in clusters beneath the fruiting pods. The pods enclose small seeds (31, 51).
- *Uses*: Traditionally, *A. gyzensis* is used in Algerian medicine to treat snakebites and scorpion stings (41). It also serves as a forage plant, contributing to dietary diversification for animals. Zeghoud et al. (47) confirmed its analgesic, antiseptic, and anti-inflammatory effects. Tedjani et al. (52) documented its use in treating diabetes, infertility, digestive disorders, angina, respiratory diseases, cancer, gum inflammation, back pain, jaundice, uterine cleansing, and as a galactagogue. It is further valued as a fortifying remedy.

##### 4.2.1.5 Chemical composition of *Astragalus* species of the septentrional Algerian Sahara

**Figure 6:**
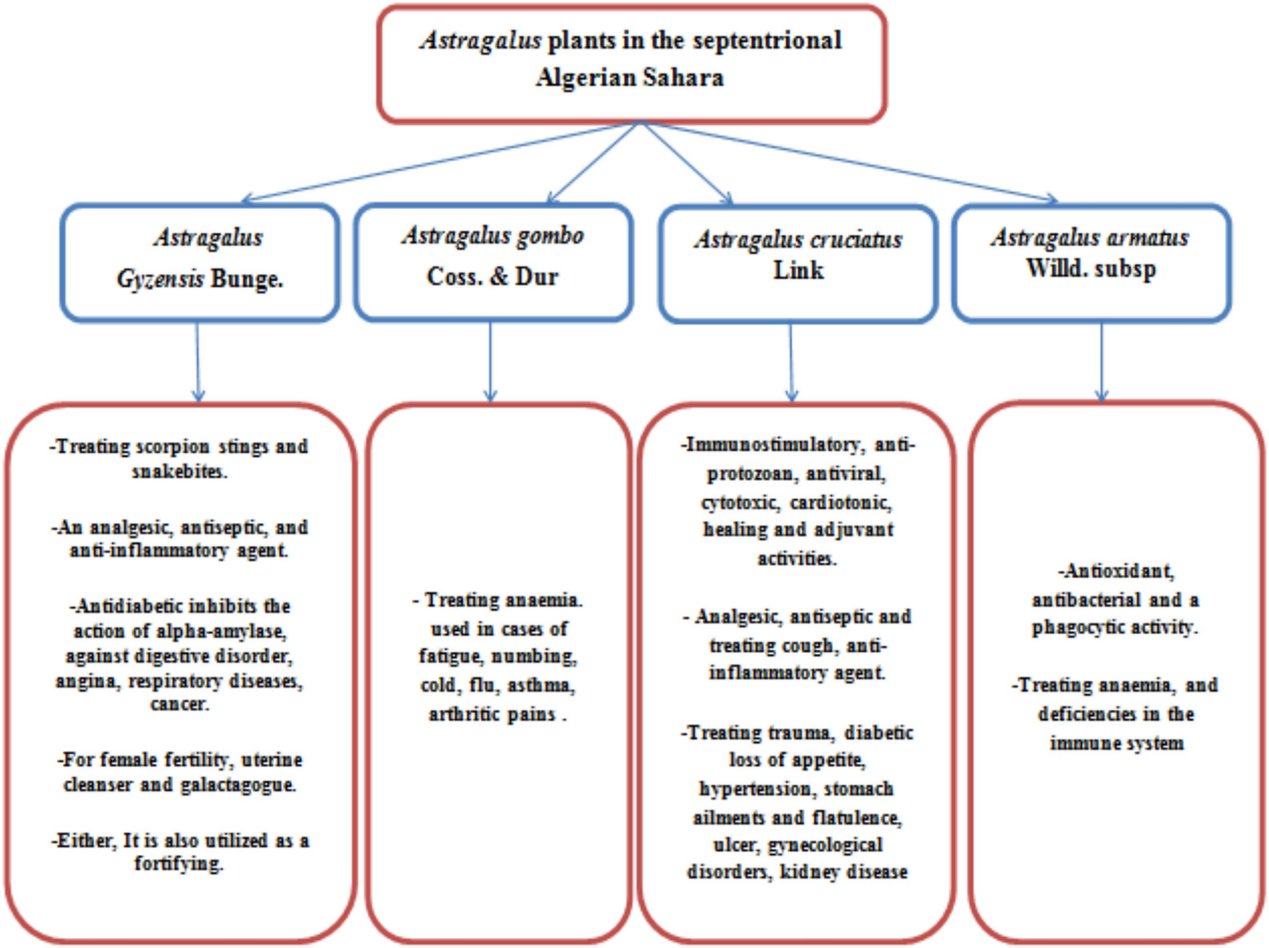
Uses and some activities of *Astragalus* plants in the septentrional Algerian Sahara.

The genus Astragalus is chemically diverse yet exhibits a high degree of uniformity, containing six main classes of bioactive compounds: indolizidine alkaloids, nitro compounds, seleniferous derivatives, polysaccharides, saponins, and flavonoids (53) (Figure 6).

###### 4.2.1.5.1 Toxic compounds: indolizidine alkaloids, nitro compounds, and seleniferous derivatives

*Astragalus cruciatus* is commonly consumed by camels when fresh, but overgrazing under extreme heat conditions can induce a syndrome locally referred to as “Asaydal,” characterized by digestive and neurological disorders. In its dried state, particularly in the seeds, the plant becomes highly toxic and can lead to the often fatal condition “l-gergâr,” which manifests with more severe symptoms than Asaydal (46). This toxicity is primarily attributed to nitro compounds present in the leaflets, especially in species with simple trichomes such as *A. cruciatus* (38). These nitro compounds have been increasingly used as chemotaxonomic markers to differentiate nitro-bearing from nitro-free *Astragalus* species.

Similarly, Tedjani et al. (48) highlighted the potential toxicity of *A. gyzensis* in both traditional medicine and forage, stressing that its therapeutic use should be carefully monitored. Inappropriate or uncontrolled applications have been linked to poisoning incidents in both humans and animals (Table 1).

**Table 1:** Therapetic uses of *Astragalus* plants in the septentrional Algerian Sahara.

Species (local name)	Reported major bioactive compound(s)	Reported biological/therapeutic effects	Source
*A. gombo* Coss. & Dur. (Feila)	Polysaccharides (WSP, galactomannan)—WSP yield 6.8%; mannose/galactose ratio ~1.7; 1.1 × 10^6^ g·mol^−1^. Also reported high cellulose content: cellulose yield 54% (seed)	Antidiabetic activity (α-amylase inhibition); antioxidant (modest); potential industrial use as cellulose source; mucilage with rheological applications	(74, 77)
*A. armatus* Willd. (Katad)	Saponins: cycloartane-type triterpene saponins (e.g., armatoside I & II; trigonoside II, trojanoside H). Flavonoids: isorhamnetin derivatives—n-butanol fraction contained one isorhamnetin triglycoside detected at ~20% (major quantity) in that fraction. Polysaccharide (WSPF, galactomannan-rich)—Man:Gal ≈ 1.13.	Immunostimulatory/phagocytosis stimulation (carbon clearance increase)—probably saponin-related; antioxidant activity (flavonoids, polysaccharides); moderate AChE/BChE inhibition for ethyl acetate extracts (weak). Galactomannan: anti-complementary and antioxidant activities	(34, 58, 76)
*A. cruciatus* Link. (Bouakifa)	Saponins: azukisaponin V, astragaloside VIII isolated from methanol extract. Nitro compounds/nitrotoxins—species reported to contain nitro-compounds (seed/leaf). Various flavonoids	Traditional uses: analgesic, antiseptic, anti-inflammatory; used for trauma, digestive disorders, gynecological disorders, hypertension, scorpion/snake bites, fertility, and cosmetics. Toxic to livestock when overgrazed (causes Asaydal/l-gergâr)—linked to nitrotoxins concentrated in seeds	(38, 52)
*A. gyzensis* Bunge. (Dlilia)	Polysaccharide (mucilage/galactomannan type)—identified by TLC and FT-IR as galactomannan.	Ethnomedical uses: treatment of scorpion and snake stings; analgesic, antiseptic, anti-inflammatory. Reported uses against diabetes, digestive disorders, respiratory disease, uterine/gynecological uses. Caution advised for toxicity when used improperly	(48)

Phytochemical investigations have confirmed that more than 470 *Astragalus* species synthesize nitro compounds, particularly 3-nitro-1-propyl-*β*-D-glucopyranoside. A large-scale screening in Iran revealed nitrotoxin production in 16 out of 37 tested species, including *A. remotifolius*, *A. ammodendroides*, *A. florulentus*, *A. angustiflorus*, *A. apricus*, *A. maymanensis*, *A. daenensis*, *A. campylotrichus*, *A. robustus*, *A. elegans*, *A. macrostachys*, and others, with toxic metabolite levels ranging between 4 and 25 mg NO₂/g plant (54). From *A. sikokianus* root extracts, four aliphatic nitro compounds and *p*-hydroxybenzoic acid were isolated, including glucosides of 3-nitropropanoic acid such as corynocarpine and karakin (20).

In addition, indolizidine alkaloids such as swainsonine have been reported (55), alongside two unique caprolactam alkaloids—3-(dimethylamino)hexahydro-2H-azepin-2-one and 3-(methylamino)-hexahydro-2H-azepin-2-one—identified from *A. cryptanthus* (55).

Another toxicological concern is selenium accumulation. Astragalus is the largest genus of selenium hyperaccumulators, with at least 25 recognized species able to accumulate up to 0.6% selenium of their dry weight—100–1,000 times higher than non-accumulators growing in the same soil (56). Notable seleniferous species include *A. bisulcatus*, *A. saurinus*, *A. praelongus*, *A. flavus*, and *A. tenellus* (53).

###### 4.2.1.5.2 Saponins

Saponins constitute one of the most extensively studied classes of bioactive compounds in *Astragalus* species (57).

The *n*-butanol extract of *A. armatus* pods revealed the presence of a cycloartane-type saponin (4R-cycloartan-1*α*, 3*β*, 7*β*, 24,25-pentaol-3-O-α-L-rhamnopyranosyl-24-O-β-D-xylopyranoside), which demonstrated stimulation of phagocytic activity in the reticuloendothelial system (58). Further studies identified two acylated tridesmosidic saponins (armatosides I and II) and two cycloartane-type glycosides (trigonoside II and trojanoside H) from *A. armatus* roots (59).

From *A. cruciatus*, azukisaponin V and astragaloside VIII were isolated (38), while other investigations across the genus have reported both cycloartane-type and oleanane-type triterpenoid saponins (60). These compounds exhibit a broad spectrum of pharmacological effects, notably immunostimulatory, antiviral, anti-protozoan, cytotoxic, cardiotonic, wound-healing, and adjuvant activities (61, 62).

Notably, astragaloside VII and related analogs enhance vaccine adjuvanticity by promoting Th1/Th2 immune balance and stimulating cytokines such as IL-1*β* and IL-12 (63). Turkish *Astragalus* species have yielded 13 cycloartane-type and one oleanane-type saponin, all showing strong IL-2-inducing activity (64). Moreover, *A. membranaceus* saponins have demonstrated anticancer activity by inhibiting proliferation and invasion of gastric cancer BGC-823 cells while promoting apoptosis (65).

###### 4.2.1.5.3 Phenolic compounds

Since 2000, a substantial number of Astragalus secondary metabolites have been identified as flavonoids, including flavones, flavonols, isoflavonoids, and related derivatives (66). Flavonols such as quercetin and kaempferol (and their glycosides) are the most prevalent.

For instance, a novel acylated flavonol triglycoside, astrarmatuside, was isolated from the aerial parts of *A. armatus* (34), along with seven known flavonol glycosides. Similarly, *A. cruciatus* yielded narcissin, nicotiflorin, and additional kaempferol/isorhamnetin derivatives (38). Extracts of *A. armatus* pods showed antioxidant, antibacterial, and phagocytic activity, largely attributed to isorhamnetin glycosides (58).

Comprehensive phytochemical studies across the genus have identified over 30 flavonol glycosides from *A. caprinus* alone, primarily derivatives of quercetin and kaempferol (67). Additional unique flavonoids and glycosides have been reported in *A. vogelii*, *A. eremophilus*, *A. corniculatus*, *A. hamosus*, *A. monspessulanus*, and *A. membranaceus* (68–72). Collectively, these compounds display antioxidant, enzyme-inhibitory, and potential therapeutic properties.

###### 4.2.1.5.4 Polysaccharides

Many *Astragalus* species exude gums when stressed, composed primarily of water-soluble polysaccharides with minor protein residues (1–3%). These gums are approved as stabilizers and thickeners in food and pharmaceuticals due to their safety and non-absorption in the gastrointestinal tract (73).

Of particular interest are cellulose and galactomannans. *A. gombo* seeds were recently shown to yield cellulose at 54%, indicating their potential as an eco-friendly cellulose source (74). Likewise, *A. armatus* roots have been evaluated for paper production, while cellulose from its pods has been characterized as an effective adsorbent of methylene blue (75).

Galactomannans are widespread in the seeds of *Astragalus*. Tedjani et al. (48) reported that *A. gyzensis* polysaccharides inhibit *α*-amylase and display modest antioxidant activity, confirming their galactomannan structure. Water-soluble polysaccharide fractions (WSPF) from *A. armatus* showed anti-complementary activity (IC₅₀ = 3.907 mg/mL) and antioxidant capacity (76). Similarly, galactomannans have been extracted from *A. gombo* (77), *A. sericeocanus* (78), *A. danicus* (79), *A. cicer* (80), *A. alpinus*, and *A. tibetanus* (81), with mannose:galactose ratios typically ranging from 1:1.3 to 1:1.7 and molecular weights between 472 and 1,549 kDa.

In addition, *A. membranaceus* roots yield numerous polysaccharides (APS), extensively studied for immunomodulatory, antioxidant, antitumor, antidiabetic, hepatoprotective, and anti-inflammatory effects (82–87). Structural diversity is significant, with glucans, heteropolysaccharides, and hemicelluloses all represented. Recent studies confirm that APS stimulate innate and adaptive immune responses by activating macrophages, dendritic cells, NK cells, and T/B lymphocytes, thereby promoting cytokine and chemokine production (85).

## 5 Research gaps and future directions

This systematic review reveals several critical gaps:

- Lack of *in vivo* and clinical studies: Current evidence is largely restricted to ethnobotanical surveys and *in vitro* assays. No controlled clinical trials on Algerian *Astragalus* species were identified.
- Standardization challenges: Variability in plant parts, extraction methods, and geographic origins complicates reproducibility.
- Conservation concerns: Endemic *Astragalus* species face threats from desertification, overgrazing, and unsustainable collection. Conservation strategies must be prioritized to preserve genetic diversity and ensure sustainable utilization.
- Translational barriers: While traditional uses highlight therapeutic potential, standardizing dosage, toxicity evaluation, and bioavailability remains a significant hurdle before pharmacological application.

## 6 Conclusion and future perspective

This comprehensive review of *Astragalus* species in the Septentrional Algerian Sahara highlights their remarkable adaptability to arid and saline environments and underscores their potential as a valuable natural resource in food, aromatics, and, most importantly, medicine. The genus displays a unique phytochemical profile rich in saponins, phenolic compounds, and polysaccharides, which collectively exhibit diverse pharmacological activities ranging from immunomodulatory and antioxidant to cytotoxic and hepatoprotective effects. Our synthesis of the literature has provided a systematic overview of the distribution, traditional uses, and chemical composition of key species, thereby bridging an important knowledge gap, particularly for lesser-known taxa within this genus.

Despite these promising findings, our study should be regarded as an initial step. Significant research gaps remain, particularly in the translation of *in vitro* and *in vivo* observations into clinically validated outcomes. The biological activities attributed to *Astragalus* metabolites—such as the immunostimulatory effects of saponins, the antioxidant potential of flavonoids, and the bioactivity of galactomannans—require further exploration through detailed mechanistic studies, pharmacokinetic profiling, and ultimately, well-designed clinical trials. Moreover, the toxicological risks associated with nitro compounds, alkaloids, and selenium hyperaccumulation demand careful toxicological evaluations to ensure safety in both medicinal and dietary applications.

Future research should also address conservation strategies and sustainable harvesting practices, given the ecological importance of these species and their adaptation to fragile desert ecosystems. Advances in biotechnology, such as metabolic engineering and polysaccharides modification, could further enhance the therapeutic and industrial potential of Astragalus resources.

In conclusion, the *Astragalus* species of the Septentrional Algerian Sahara represent a fascinating yet underexplored domain in natural product research. Their resilience and biochemical richness position them as promising candidates for future pharmacological, ethnobotanical, and ecological studies. It is anticipated that the present review will serve as a catalyst for deeper investigations, unlocking the full medicinal and economic potential of these remarkable desert plants while ensuring their sustainable utilization.

## Data Availability

The original contributions presented in the study are included in the article/supplementary material, further inquiries can be directed to the corresponding author.
